# Hyperpolarized ^3^He and ^129^Xe magnetic resonance imaging apparent diffusion coefficients: physiological relevance in older never‐ and ex‐smokers

**DOI:** 10.14814/phy2.12068

**Published:** 2014-07-17

**Authors:** Miranda Kirby, Alexei Ouriadov, Sarah Svenningsen, Amir Owrangi, Andrew Wheatley, Roya Etemad‐Rezai, Giles E. Santyr, David G. McCormack, Grace Parraga

**Affiliations:** 1 Imaging Research Laboratories, Robarts Research Institute, The University of Western Ontario, London, Ontario, Canada; 2 Department of Medical Biophysics, The University of Western Ontario, London, Ontario, Canada; 3 Graduate Program in Biomedical Engineering, The University of Western Ontario, London, Ontario, Canada; 4 Department of Medical Imaging, The University of Western Ontario, London, Ontario, Canada; 5 Division of Respirology, Department of Medicine, The University of Western Ontario, London, Ontario, Canada

**Keywords:** Apparent diffusion coefficient, COPD, emphysema, hyperpolarized ^3^He MRI, hyperpolarized ^129^Xe MRI

## Abstract

Noble gas pulmonary magnetic resonance imaging (MRI) is transitioning away from ^3^He to ^129^Xe gas, but the physiological/clinical relevance of ^129^Xe apparent diffusion coefficient (ADC) parenchyma measurements is not well understood. Therefore, our objective was to generate ^129^Xe MRI ADC for comparison with ^3^He ADC and with well‐established measurements of alveolar structure and function in older never‐smokers and ex‐smokers with chronic obstructive pulmonary disease (COPD). In four never‐smokers and 10 COPD ex‐smokers, ^3^He (*b* = 1.6 sec/cm^2^) and ^129^Xe (*b* = 12, 20, and 30 sec/cm^2^) ADC, computed tomography (CT) density‐threshold measurements, and the diffusing capacity for carbon monoxide (DL_CO_) were measured. To understand regional differences, the anterior–posterior (AP_G_) and superior–inferior (∆SI) ADC differences were evaluated. Compared to never‐smokers, COPD ex‐smokers showed greater ^3^He ADC (*P* = 0.006), ^129^Xe ADC_b12_ (*P* = 0.006), and ADC_b20_ (*P* = 0.006), but not for ADC_b30_ (*P* > 0.05). Never‐smokers and COPD ex‐smokers had significantly different AP_G_ for ^3^He ADC (*P* = 0.02), ^129^Xe ADC_b12_ (*P* = 0.006), and ADC_b20_ (*P* = 0.01), but not for ADC_b30_ (*P* > 0.05). ∆SI for never‐ and ex‐smokers was significantly different for ^3^He ADC (*P* = 0.046), but not for ^129^Xe ADC (*P* > 0.05). There were strong correlations for DL_CO_ with ^3^He ADC and ^129^Xe ADC_b12_ (both *r* = −0.95, *P* < 0.05); in a multivariate model ^129^Xe ADC_b12_ was the only significant predictor of DL_CO_ (*P* = 0.049). For COPD ex‐smokers, CT relative area <−950 HU (RA_950_) correlated with ^3^He ADC (*r* = 0.90, *P* = 0.008) and ^129^Xe ADC_b12_ (*r* = 0.85, *P* = 0.03). In conclusion, while ^129^Xe ADC_b30_ may be appropriate for evaluating subclinical or mild emphysema, in this small group of never‐smokers and ex‐smokers with moderate‐to‐severe emphysema, ^129^Xe ADC_b12_ provided a physiologically appropriate estimate of gas exchange abnormalities and alveolar microstructure.

## Abstract (precis)

We aimed to better understand how ^129^Xe MRI measurements might explain well‐established measurements of emphysema, and explored different ^129^Xe MRI apparent diffusion coefficients (ADCs) in a small group of older never‐smokers and ex‐smokers with chronic obstructive pulmonary disease (COPD). In the same visit, ^3^He ADC, the diffusing capacity of carbon monoxide and computed tomography (CT) emphysema measurements were acquired. In this small group of older never‐smokers and ex‐smokers with COPD, ^129^Xe ADCb12 provided a physiologically appropriate estimate of gas exchange abnormalities and alveolar microstructure.

## Introduction

Magnetic resonance imaging (MRI) using hyperpolarized noble gases ^3^He and ^129^Xe provides high spatial and temporal resolution images of pulmonary gas distribution (Kauczor et al. 1997; de Lange et al. 1999; Moller et al. 2002). Parenchymal microstructure can also be probed using diffusion‐weighted (DW) imaging. DW imaging takes advantage of the gases rapid molecular diffusion to generate ^3^He and ^129^Xe apparent diffusion coefficients (ADCs; Saam et al. 2000; Salerno et al. 2002). Although the first demonstrations of inhaled hyperpolarized gas MRI used ^129^Xe gas (Albert et al. 1994; Mugler et al. 1997), until recently, most examinations in subjects with chronic obstructive pulmonary disease (COPD) used ^3^He gas mainly due to the nearly threefold higher gyromagnetic ratio and higher polarizations – both of which independently contribute to greater image quality for ^3^He MRI. These previous studies showed that ^3^He ADC values are reproducible in emphysematous subjects (Morbach et al. 2005; Diaz et al. 2008; Mathew et al. 2008), sensitive to lung microstructure and airspace size (Saam et al. 2000; Salerno et al. 2002), and correlate with spirometry (Salerno et al. 2002; Diaz et al. 2009), diffusing capacity of carbon monoxide (DL_CO_; Fain et al. 2006), and x‐ray computed tomography (CT) measurements of emphysema (Diaz et al. 2009). Importantly, ^3^He MRI ADC measurements were directly compared with stereological measurements of fixed lung tissue and this showed the high sensitivity of ^3^He MRI ADC to lung surface area abnormalities in emphysematous tissue (Woods et al. 2006). Despite these important findings, ^3^He MRI is unlikely to be used clinically because of its relatively high cost and low abundance (Fain et al. 2010). On the other hand, ^129^Xe gas is substantially more abundant, relatively inexpensive, and clinical quantities may be polarized in 30–60 min (Mugler et al. 1997, 2004). Because of this, there is considerable interest in transitioning to ^129^Xe MRI.

Although ^129^Xe gas is capable of transmembrane diffusion (Cleveland et al. 2010) this leads to adverse systemic effects when used in high concentrations; Xe has been widely and safely used in pulmonary CT (Chae et al. 2008; Honda et al. 2012), cerebral blood flow imaging (Carlson et al. 2011), and lung scintigraphy (Suga 2002). Recent ^129^Xe MRI safety studies demonstrated that the gas concentrations compatible with MRI are well tolerated in healthy subjects and those with respiratory disease (Driehuys et al. 2012; Shukla et al. 2012). Previous work showed that ^129^Xe ADC (*b* = 12 sec/cm^2^; Kaushik et al. 2011) and ^129^Xe ADC morphometry measurements (Ouriadov et al. 2013) provide a way to distinguish between age‐matched subjects with and without COPD. Moreover, although strong and significant correlations for ^3^He ADC with ^129^Xe ADC (*b* = 12 sec/cm^2^) were reported (Kirby et al. 2012d), significantly lower ^129^Xe as compared to ^3^He MRI ventilation was observed in COPD ex‐smokers (Kirby et al. 2013) and asthmatics (Svenningsen et al. 2013). Regions of diminished ^129^Xe as compared to ^3^He ventilation are spatially related to emphysematous regions (Kirby et al. 2013) in COPD subjects.

It is important to consider the different self‐diffusion coefficients of ^3^He and ^129^Xe gases. For example, ^129^Xe gas has a much lower diffusion coefficient than ^3^He and, therefore, in order to achieve sufficient diffusion weighting for ^129^Xe MRI to derive meaningful lung microstructural information, appropriately large *b* values and diffusion times must be applied (Sukstanskii and Yablonskiy 2012). However, increasing the diffusion weighting is coupled with a reduction in image signal‐to‐noise ratio (SNR); therefore it is important to experimentally demonstrate the ^129^Xe ADC *b* value that provides the optimal balance between sensitivity to the lung microstructural abnormalities and feasibility with respect to image SNR. It is also essential to investigate the different ^129^Xe MRI ADC *b* values and their associations with other emphysema measurements in order to better understand how imaging estimates are related to physiological measurements in COPD patients. Previous studies have focused on ^129^Xe ADC generated with a *b* value of 12 sec/cm^2^ (Kaushik et al. 2011; Kirby et al. 2012d), and therefore here we aimed to (i) determine whether ^129^Xe ADC generated using *b* values of 12, 20, and 30 sec/cm^2^ and contemporaneously acquired ^3^He ADC (Kirby et al. 2013), distinguishes older never‐smokers from ex‐smokers with COPD, (ii) compare ^3^He and ^129^Xe ADC in the superior–inferior (SI) and anterior–posterior (AP) lung regions to better understand potential dependencies for *b* value with regional ADC, and (iii) determine the significant associations of ^129^Xe ADC with *b* values of 12, 20, and 30 sec/cm^2^ with DL_CO_ and CT measurements of emphysema, similar to those recently used in the COPDGene (Regan et al. 2010), CanCOLD (Bourbeau and Saad 2013), ECLIPSE (Vestbo and Anderson 2008), and SPIROMICS (Couper et al. 2014) studies.

## Materials and Methods

### Subjects

All subjects provided written informed consent to the study protocol that was approved by the local research ethics board and Health Canada. COPD subjects were ex‐smokers with a smoking history of at least 10 pack‐years between 50 and 85 years of age and categorized according to the Global Initiative for Chronic Lung Disease (2013) (GOLD) spirometry criteria. Healthy older never‐smokers (<1 pack‐year over their lifetime and no smoking in last 20 years) were enrolled who had no history of previous chronic or current respiratory disease.

### Pulmonary function tests

A body plethysmograph (MedGraphics Corporation, Saint Paul, MN) with the attached gas analyzer was used to measure airflow, lung volumes, and DL_CO_ according to ATS/ERS guidelines (Macintyre et al. 2005; Miller et al. 2005; Wanger et al. 2005).

### Image acquisition

Magnetic resonance imaging was performed on a whole body 3.0 Tesla Discovery 750MR (General Electric Health Care, Milwaukee, WI) MRI system with broadband imaging capability. Subjects were instructed to inhale a gas mixture from a 1.0L Tedlar^®^ bag (Jensen Inert Products, Coral Springs, FL) from functional residual capacity (FRC) and image acquisition was performed under breath‐hold conditions (Parraga et al. 2007).

Hyperpolarized ^3^He MRI was enabled using a linear bird‐cage transmit/receive chest coil (RAPID Biomedical GmbH, Wuerzburg, Germany; Farag et al. 2012). A turn‐key system (HeliSpin^™^, Polarean Inc, Durham, NC) was used to polarize ^3^He gas to 30–40% and doses (5 mL/kg body weight) diluted with N_2_ were administered in 1.0L Tedlar^®^ bags. Hyperpolarized ^3^He MRI diffusion‐weighted imaging was performed using a 2D multislice fast gradient‐recalled echo sequence (FGRE; 14 sec total data acquisition, repetition time [TR]/echo time [TE]/flip angle = 7.6 msec/3.7 msec/8°, field of view [FOV] = 40 × 40 cm, matrix 128 × 128, seven slices, 30 mm slice thickness, 0 gap) during breath hold (Parraga et al. 2007) for acquisition of two interleaved images with and without additional diffusion sensitization with *b* = 1.6 sec/cm^2^ (maximum gradient amplitude [*G*] = 1.94 G/cm, rise and fall time = 0.5 msec, gradient duration = 0.46 msec, diffusion time = 1.46 msec).

Hyperpolarized ^129^Xe MRI was enabled using a custom‐made, quadrature‐asymmetric bird‐cage coil model tuned to 35.34 MHz, as described previously (Farag et al. 2012). The XeBox‐E10 polarizer system (XeBox‐E10; Xemed LLC, Durham, NH) was used to polarize the ^129^Xe gas (86% enriched) to 10–60%. In contrast to the ^3^He gas doses which were determined according to the subjects’ body weight, for ^129^Xe MRI a 50/50 mixture of ^129^Xe and ^4^He gas was obtained by dispensing ^129^Xe directly into 1.0L Tedlar^®^ bags prefilled with ^4^He. Polarization of the diluted dose was quantified with use of a Polarimeter (Polarean, Durham, NC). ^129^Xe MRI DW images were obtained using a 2D multislice FGRE sequence; two interleaved images (16 sec total data acquisition, TE/TR/flip angle = 9.8 msec/11.0 msec/5°, bandwidth = 31.25 kHz, FOV = 40 × 40 cm, matrix 128 × 80, seven slices, 30 mm slice thickness, 0 gap) with and without additional diffusion sensitization with *b* = 12 sec/cm^2^ (*G* = 2.90 G/cm, rise and fall time = 0.5 msec, gradient separation = 2.0 msec, gradient duration = 2.0 msec, diffusion time = 5 msec), *b* = 20 sec/cm^2^ (*G* = 3.75 G/cm, rise and fall time = 0.5 msec, gradient separation = 2.0 msec, gradient duration = 2.0 msec, diffusion time = 5 msec), and *b* = 30 sec/cm^2^ (*G* = 4.60 G/cm, rise and fall time = 0.5 msec, gradient separation = 2 msec, gradient duration = 2.0 msec, diffusion time = 5 msec) were acquired in separate image acquisitions, as described previously (Kirby et al. 2012d; Boudreau et al. 2013).

Computed tomography was performed on a 64‐slice Lightspeed VCT scanner (GEHC, Milwaukee, WI). A single spiral acquisition of the entire lung was acquired from apex to base with subjects in breath‐hold condition after inhalation of N_2_ from a 1.0L Tedlar^®^ bag (detector configuration of 64 × 0.625 mm, 120 kVp, 100 effective mA, tube rotation time of 500 msec, and a pitch of 1.0). Reconstruction of the data was performed using a standard convolution kernel and a slice thickness of 1.25 mm.

### Image analysis

^3^He ADC and ^129^Xe ADC with *b* values of 12 sec/cm^2^ (ADC_b12_), 20 sec/cm^2^ (ADC_b20_), and 30 sec/cm^2^ (ADC_b30_) were generated as described previously (Kirby et al. 2012b). Briefly, we first segmented the ^3^He MRI non‐DW images using k‐means cluster algorithm as described previously for ^3^He MRI segmentation (Kirby et al. 2012a). The segmented binary mask for the non‐DW images was applied to the DW images. The ADC maps were generated on a voxel‐by‐voxel basis according to equation 1: 
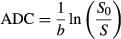


where *b* = 1.6 sec/cm^2^, *S*_0_ = the segmented non‐DW image, and *S* = the diffusion‐weighted image. The regional differences in ADC were evaluated for all AP coronal slices and for each coronal slice in three SI regions of interest (ROIs). The AP gradient (AP_G_) was defined as the slope of the line of best fit that described the change in ADC as a function of distance in centimeters for all slices in the anterior to posterior direction. The three SI ROI were generated by dividing the center coronal slice into three equivalent ROIs from apex to base and applying those boundaries to all slices. ∆SI was the mean change in ADC in the superior and inferior ROI. To compare ^3^He and ^129^Xe ADC ∆SI and AP_G_ to better understand any dependency on *b* value for regional ADC differences in the respective gases, we compared the older never‐smokers to the COPD subjects, as well as to only the COPD ex‐smokers with emphysema. COPD subjects without emphysema are unlikely to show an increased ^3^He or ^129^Xe ADC ∆SI and AP_G_ difference compared to the older never‐smokers. The SNR for ^3^He and ^129^Xe DW images and nondiffusion‐weighted (NDW) images were determined by calculating the mean voxel value within a 5 × 5 voxel ROI for four representative ROI within the lung parenchyma, and dividing by the standard deviation of the mean voxel values for four representative 5 × 5 voxel ROI within the image background where there was no lung structure, for each slice. SNR was determined for each slice and then averaged to obtain a single SNR value for each subject image.

Quantitative CT measurements were performed using the Pulmonary Workstation 2.0 (VIDA Diagnostics, Inc., Coralville, IA); the relative area with attenuation values below −950 Hounsfield Units (HU; RA_950_), −910 HU (RA_910_), and −856 HU (RA_856_) were generated (Gevenois et al. 1996) as well as the CT HU value at which 15% of the voxels in the frequency distribution histogram have a lower density (HU_15%_). A CT density threshold for emphysema of RA_950_ > 6.8% was adopted based on a previous study that reported RA_950_ = 6.8% as the upper 95% limit of predicted normal values (Gevenois et al. 1996).

Qualitative visual scoring was also performed by an expert chest radiologist (R. E.‐R.) using a method adapted from Bankier et al. (1996). Emphysema scores were based on the percentage of low attenuation, tissue destruction, and vascular disruption.

### Statistical methods

A Mann–Whitney unpaired two‐tailed *t*‐test was performed for statistical comparison of all pulmonary function measurements, ^3^He and ^129^Xe ADC, ^3^He and ^129^Xe ADC AP_G_, and ^3^He and ^129^Xe ADC SI using GraphPad Prism version 4.00 (GraphPad Software Inc., San Diego, CA). A one‐way analysis of variance (ANOVA) was used for statistical comparison of the mean SNR values for ADC DW images for ^3^He and ^129^Xe with all three *b* values using IBM SPSS Statistics 20.0 (SPSS Inc., Chicago, IL). Linear regression (*r*^2^) and Pearson correlation coefficients (*r*) were used to determine the relationships between imaging and other measurements using GraphPad. A Holm–Bonferroni correction (Van Bell et al. 2004) was used to correct for multiple tests. Multivariate linear regression modeling was used to evaluate the relationship between DL_CO_ and ^3^He ADC, ^129^Xe ADC_b12_, ^129^Xe ADC_b20_, and ^129^Xe ADC_b30_ as well as between CT density measurements and ^3^He ADC, ^129^Xe ADC_b12_, ^129^Xe ADC_b20_, and ^129^Xe ADC_b30_ using PROC REG in SAS ^®^ 9.2 Software (SAS Institute Inc., Cary, NC). In all statistical analyses, results were considered significant when the probability of making a Type I error was <5% (*P* < 0.05).

## Results

### Study subjects and pulmonary function

Table 1 shows all demographic and pulmonary function measurements for four older never‐smokers and 10 COPD ex‐smokers (GOLD Class I: *n* = 1; GOLD Class II: *n* = 6; GOLD Class III: *n* = 2; GOLD Class IV: *n* = 1). Although there were no significant differences in age, sex, or BMI, the never‐smokers and COPD ex‐smokers were significantly different with respect to FEV_1_ (*P* = 0.01), FEV_1_/FVC (*P* = 0.002), TLC (*P* = 0.02), RV (*P* = 0.02), FRC (*P* = 0.01), and DL_CO_ (*P* = 0.006).

**Table 1.** Subject demographics and pulmonary function measurements

	Older never‐smokers (*n* = 4)	All COPD (*n* = 10)	COPD with emphysema (*n* = 7)
Age years (±SD)	67 (13)	74 (4)	74 (4)
Female sex	2	2	2
Weight kg (±SD)	72 (11)	77 (22)	68 (18)
BMI kg/m^2^ (±SD)	26.4 (2.8)	25.4 (5.0)	23.6 (4.6)
FEV_1_%_pred_ (±SD)	103 (6)	57 (24)*	52 (27)*
FVC%_pred_ (±SD)	102 (8)	91 (19)	92 (22)
FEV_1_/FVC (±SD)	0.76 (0.02)	0.46 (0.14)*	0.40 (0.13)*
TLC%_pred_ (±SD)	102 (8)	115 (8)*	118 (7)*
RV%_pred_ (±SD)	102 (9)	159 (46)*	167 (54)
RV/TLC (±SD)	0.39 (0.09)	0.53 (0.14)	0.55 (0.16)
IC%_pred_ (±SD)	113 (16)	85 (31)	77 (32)
FRC%_pred_ (±SD)	93 (15)	141 (35)*	154 (34)*
DL_CO_%_pred_ (±SD)	104 (12)	41 (17)*	36 (13)*

SD, standard deviation; BMI, body mass index; FEV_1_, forced expiratory volume in 1 sec; %_pred_, percent predicted; FVC, forced vital capacity; TLC, total lung capacity; RV, reserve volume; IC, inspiratory capacity; FRC, functional residual capacity; DL_CO_, carbon monoxide diffusion capacity of the lung.

* Significant difference (*P* < 0.05) between older never‐smokers and chronic obstructive pulmonary disease (COPD) subjects.

### CT measurements of emphysema

Table 2 shows mean CT density thresholds (RA_950_, RA_910_, RA_856_, HU_15%_) and CT emphysema scores for the COPD ex‐smokers, by subject. For all COPD ex‐smokers, mean RA_950_, RA_910_, RA_856_, HU_15%_, and emphysema score were 18.8 ± 14.1%, 39.6 ± 20.6%, 65.0 ± 16.0%, −948 ± 30 HU, and 1.1 ± 0.7, respectively, consistent with mild emphysema. Subjects 2 (RA_950_ = 5.3%), 4 (RA_950_ = 1.5%), and 9 (RA_950_ = 2.1%) did not have CT evidence of emphysema (RA_950_ < 6.8%). For only the COPD ex‐smokers with emphysema, mean RA_950_, RA_910_, RA_856_, HU_15%_, and visual CT scoring measurements were 25.5 ± 10.9%, 48.9 ± 15.8%, 71.1 ± 12.4%, −964 ± 16 HU, and 1.4 ± 0.6, respectively.

**Table 2.** Subject listing of ^3^He and ^129^Xe magnetic resonance imaging ADC and computed tomography (CT) measurements

Subject	ADC measurements				CT measurements				
	^3^He ADC_*b* = 1.6_ (cm^2^/sec)	^129^Xe ADC_*b* = 12_ (cm^2^/sec)	^129^Xe ADC_*b* = 20_ (cm^2^/sec)	^129^Xe ADC_*b* = 30_ (cm^2^/sec)	RA_950_ (HU)	RA_910_ (HU)	RA_856_ (HU)	HU_15%_ (HU)	Visual score
Older never‐smokers									
1	0.247	0.048	0.048	0.040	–	–	–	–	–
2	0.230	0.049	0.049	0.035	–	–	–	–	–
3	0.215	0.049	0.049	0.036	–	–	–	–	–
4	0.242	0.053	0.053	0.042	–	–	–	–	–
All (±SD)	0.223 (0.014)	0.050 (0.002)	0.044 (0.002)	0.038 (0.003)	–	–	–	–	–
Chronic obstructive pulmonary disease Ex‐Smokers									
1	0.611	0.096	0.081	–	33.33	49.35	67.78	−981	2.35
2	0.305	0.057	0.057	–	5.26	30.43	67.90	−930	0.16
3	0.531	0.087	0.078	–	32.07	65.81	85.86	−972	1.55
4	0.320	0.058	0.050	–	1.53	9.13	36.68	−895	0.43
5	0.591	0.095	0.088	–	36.83	66.19	84.28	−975	0.89
6	0.617	0.095	0.070	–	34.25	60.48	79.03	−977	–
7	0.504	0.085	0.079	0.064	12.12	31.56	60.64	−943	0.83
8	0.474	0.083	0.072	0.055	16.77	40.14	66.75	−954	1.40
9	0.283	0.056	0.046	–	2.12	13.38	47.61	−907	0.34
10	0.526	0.092	0.076	0.052	13.22	29.00	53.02	−945	1.49
All (±SD)	0.476 (0.128)	0.080 (0.017)	0.070 (0.014)	0.057 (0.006)	18.75 (14.11)	39.55 (20.56)	64.95 (15.95)	−948 (30)	1.05 (0.71)

ADC, apparent diffusion coefficient; SNR, signal‐to‐noise ratio; RA_950_, relative area with attenuation values below −950 HU; RA_910_, relative area with attenuation values below −910 HU; RA_856_, relative area with attenuation values below −856 HU; HU_15%_, the hounsfield unit value at which 15% of the voxels in the frequency distribution histogram have a lower density.

### Hyperpolarized MRI ADC

**Figure 1.**
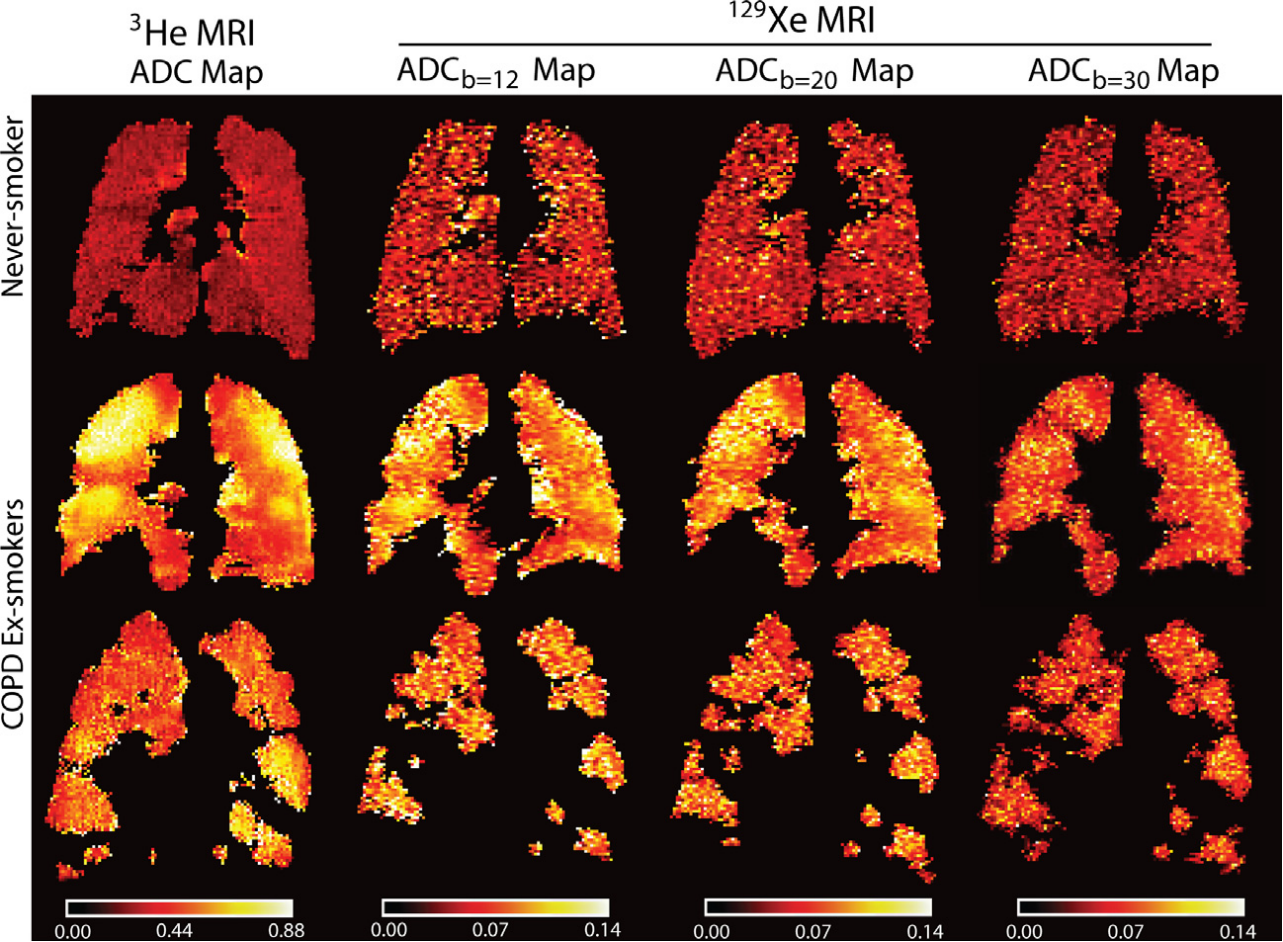
Hyperpolarized ^3^He apparent diffusion coefficient (ADC) and ^129^Xe ADC maps for a representative older never‐smoker and two chronic obstructive pulmonary disease (COPD) ex‐smokers. ^3^He ADC and ^129^Xe ADC maps with *b* value of 12 sec/cm^2^ (ADC_*b* = 12_), 20 sec/cm^2^ (ADC_*b* = 20_), and 30 sec/cm^2^ (ADC_*b* = 30_) of the coronal center slice for an older never‐smoker (79‐year‐old male, FEV_1_ = 96%_pred_, FEV_1_/FVC = 74%) and two COPD ex‐smokers (top: 71‐year‐old male, FEV_1_ = 107%_pred_, FEV_1_/FVC = 58%; bottom: 76‐year‐old male, FEV_1_ = 35%_pred_, FEV_1_/FVC = 31%).

**Figure 2.**
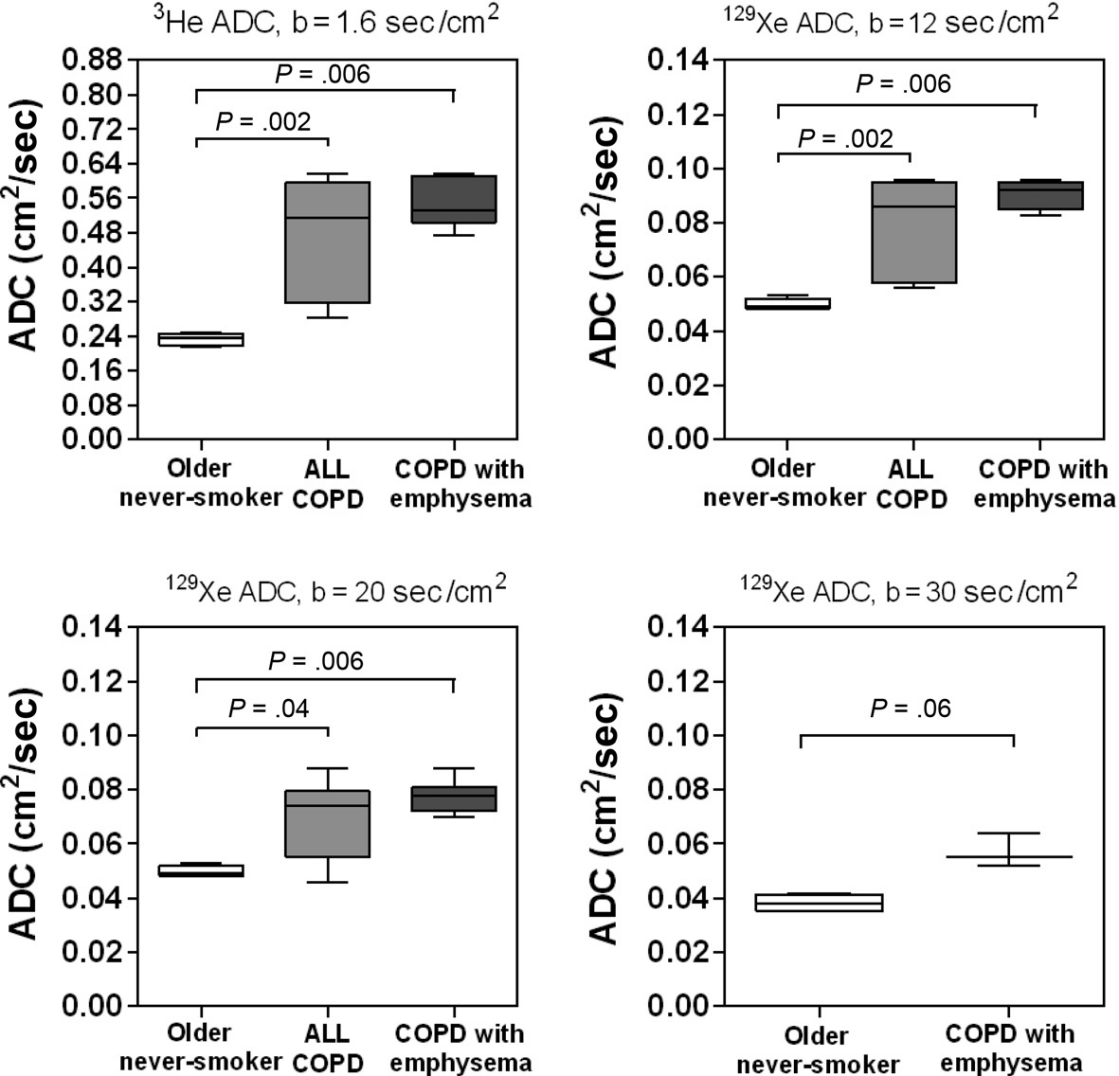
^3^He and ^129^Xe magnetic resonance imaging apparent diffusion coefficient (ADC) measurements for older never‐smokers, all chronic obstructive pulmonary disease (COPD) ex‐smokers, and COPD ex‐smokers with emphysema. Box‐and‐Whisker plot where box extends from the 25th to 75th percentiles of ADC values, the line within the box is the median ADC value and error bars are the minimum and maximum ADC values. Significance of difference (*P* < 0.05) determined using a Mann–Whitney unpaired two‐tailed *t*‐test.

Figure 1 shows the central coronal ^3^He ADC, ^129^Xe ADC_b12_, ^129^Xe ADC_b20_, and ^129^Xe ADC_b30_ maps for an older never‐smoker and two COPD ex‐smokers. ADC maps appear brighter and more heterogeneous for the COPD ex‐smokers in comparison with the healthy never‐smoker for ^3^He MRI as well as for ^129^Xe MRI for all *b* values. As shown in Figure 2, there were statistically significantly greater ADC values for all COPD ex‐smokers as well as only those COPD ex‐smokers with emphysema in comparison to the older never‐smokers for ^3^He ADC (all COPD: *P* = 0.002; COPD with emphysema: *P* = 0.006), ^129^Xe ADC_b12_ (all COPD: *P* = 0.002; COPD with emphysema: *P* = 0.006), and ^129^Xe ADC_b20_ (all COPD: *P* = 0.04; COPD with emphysema: *P* = 0.006), but not for ^129^Xe ADC_b30_ (*P* = 0.06).

For all subjects, mean SNR was statistically significantly greater for ^3^He DW images (^3^He DW SNR = 34 ± 19) in comparison with ^129^Xe DW images with *b* value = 12 sec/cm^2^ (^129^Xe DW SNR = 12 ± 7, *P* < 0.0001), *b* value = 20 sec/cm^2^ (^129^Xe DW SNR = 10 ± 7, *P* < 0.0001), and *b* value = 30 sec/cm^2^ (^129^Xe DW SNR = 4 ± 1, *P* < 0.0001), however, there was no significant difference between mean ^129^Xe DW SNR for all *b* values (*P* > 0.05). Importantly, for all subjects there was no significant correlation between SNR and ADC for ^3^He (*r* = 0.32, *P* = 0.27), ^129^Xe ADC_b12_ (*r* = 0.47, *P* = 0.09), and ^129^Xe ADC_b20_ (*r* = 0.50, *P* = 0.07), however, there was a significant correlation between SNR and ^129^Xe ADC_b30_ (*r* = 0.84, *P* = 0.02). For all subjects, mean SNR was statistically significantly greater for ^3^He NDW, or *b *=**0, images (^3^He NDW SNR = 65 ± 39) than for ^129^Xe NDW images with *b* value = 12 sec/cm^2^ (^129^Xe NDW SNR = 29 ± 21, *P* = 0.008), *b* value = 20 sec/cm^2^ (^129^Xe NDW SNR = 33 ± 26, *P* = 0.02), and *b* value = 30 sec/cm^2^ (^129^Xe NDW SNR = 18 ± 11, *P* = 0.003), however, there was no significant difference between mean ^129^Xe NDW SNR for all *b* values (*P* > 0.05).

### ADC regional differences

**Figure 3.**
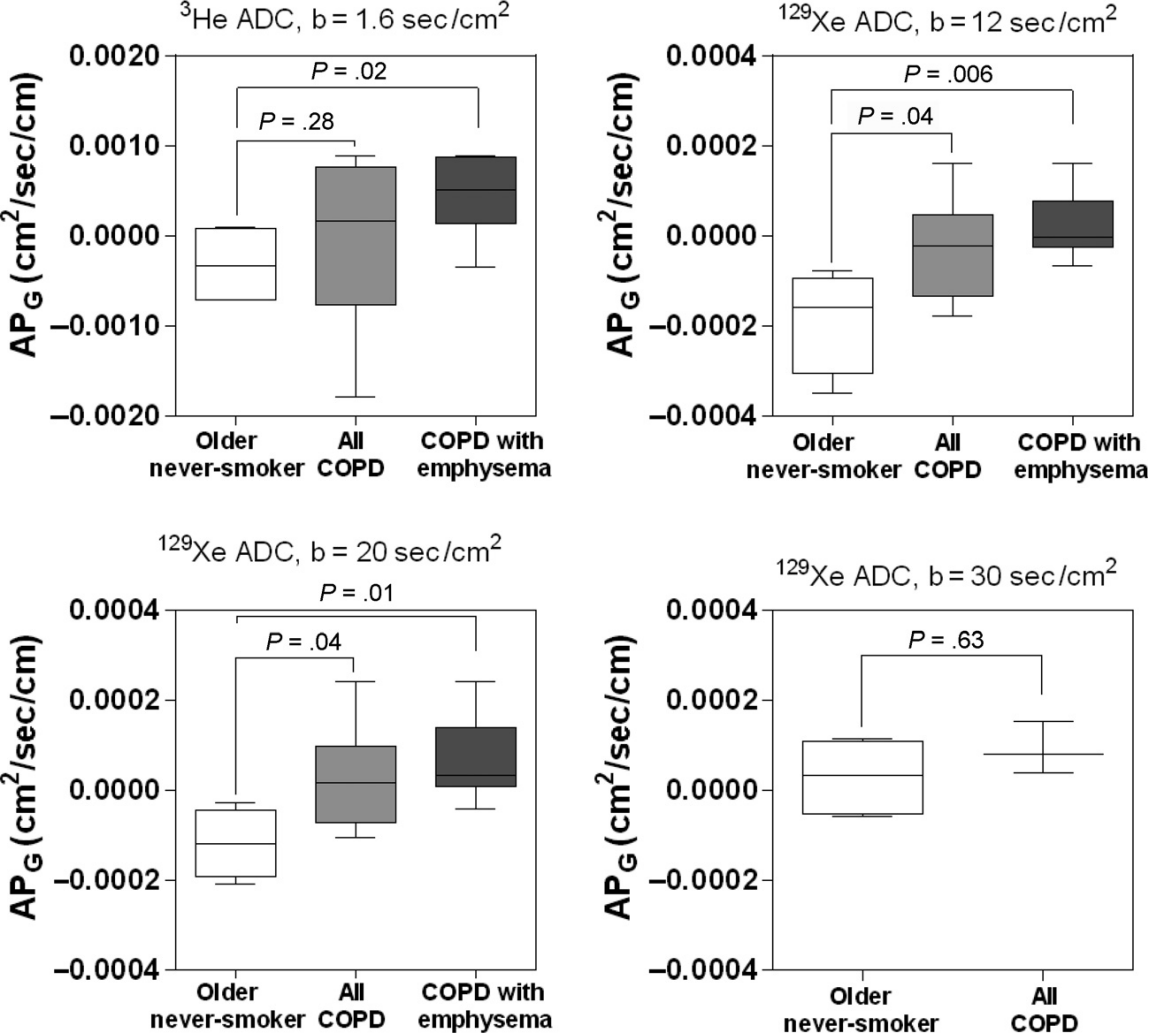
^3^He and ^129^Xe magnetic resonance imaging apparent diffusion coefficient (ADC) anterior–posterior gradients (AP_G_) for older never‐smokers, all chronic obstructive pulmonary disease (COPD) ex‐smokers, and COPD ex‐smokers with emphysema. Box‐and‐Whisker plot where box extends from the 25th to 75th percentiles of ADC values, the line within the box is the median ADC AP_G_ value and error bars are the minimum and maximum ADC AP_G_ values. Significance of difference (*P* < 0.05) determined using a Mann–Whitney unpaired two‐tailed *t*‐test.

Figure 3 shows the ^3^He and ^129^Xe MRI ADC anterior–posterior gradients (AP_G_) for older never‐smokers as well as all COPD ex‐smokers and only the COPD ex‐smokers with emphysema. There were statistically significantly greater AP_G_ for the COPD ex‐smokers than the older never‐smokers for ^129^Xe ADC_b12_ (*P* = 0.04) and ^129^Xe ADC_b20_ (*P* = 0.04), but not for ^3^He ADC (*P* > 0.05) or ^129^Xe ADC_b30_ (*P* > 0.05); there were also statistically significantly greater AP_G_ for the COPD ex‐smokers with emphysema than the older never‐smokers for ^3^He ADC (*P* = 0.02), ^129^Xe ADC_b12_ (*P* = 0.006), and ^129^Xe ADC_b20_ (*P* = 0.01), but not for ^129^Xe ADC_b30_ (*P* > 0.05).

**Figure 4.**
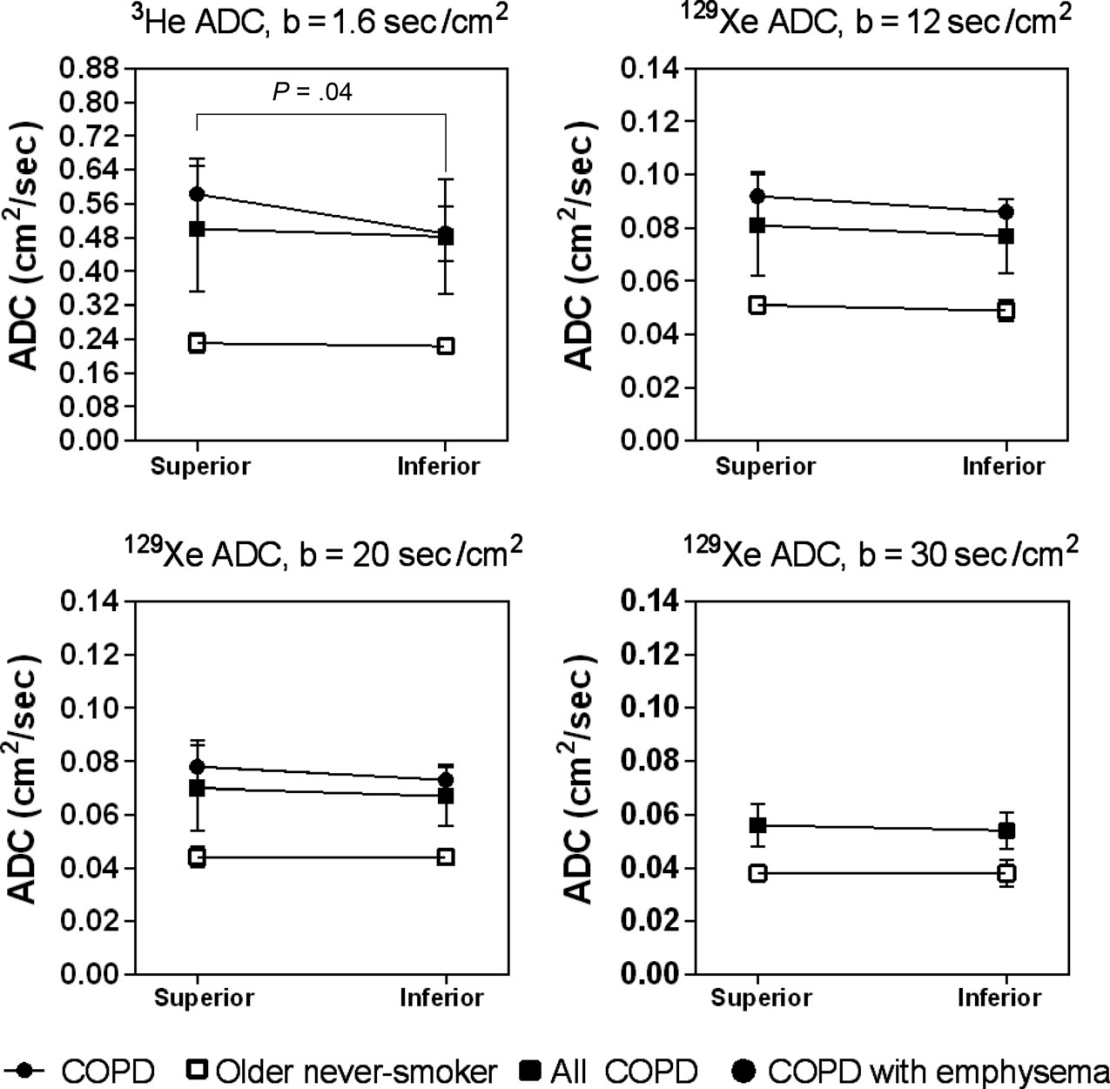
^3^He and ^129^Xe magnetic resonance imaging (MRI) apparent diffusion coefficient (ADC) for superior and inferior regions of interest in older never‐smokers, all chronic obstructive pulmonary disease (COPD) ex‐smokers, and COPD ex‐smokers with emphysema. There was a significant difference in mean ADC in the superior to inferior lung regions between the older never‐smokers and the COPD ex‐smokers with emphysema for ^3^He MRI ADC (*P* = 0.04), but there were no other significant differences. Significance of difference (*P* < 0.05) determined using a Mann–Whitney unpaired two‐tailed *t*‐test.

Figure 4 shows the ADC in the superior and inferior ROI for the older never‐smokers, all COPD ex‐smokers and only the COPD ex‐smokers with emphysema. For the COPD ex‐smokers with emphysema, mean ^3^He ADC was statistically significantly greater in the superior ROI than the inferior ROI (*P* = 0.04), however, there were no other significant SI ADC differences in ^3^He or ^129^Xe MRI.

### ADC relationships with pulmonary function and CT

**Figure 5.**
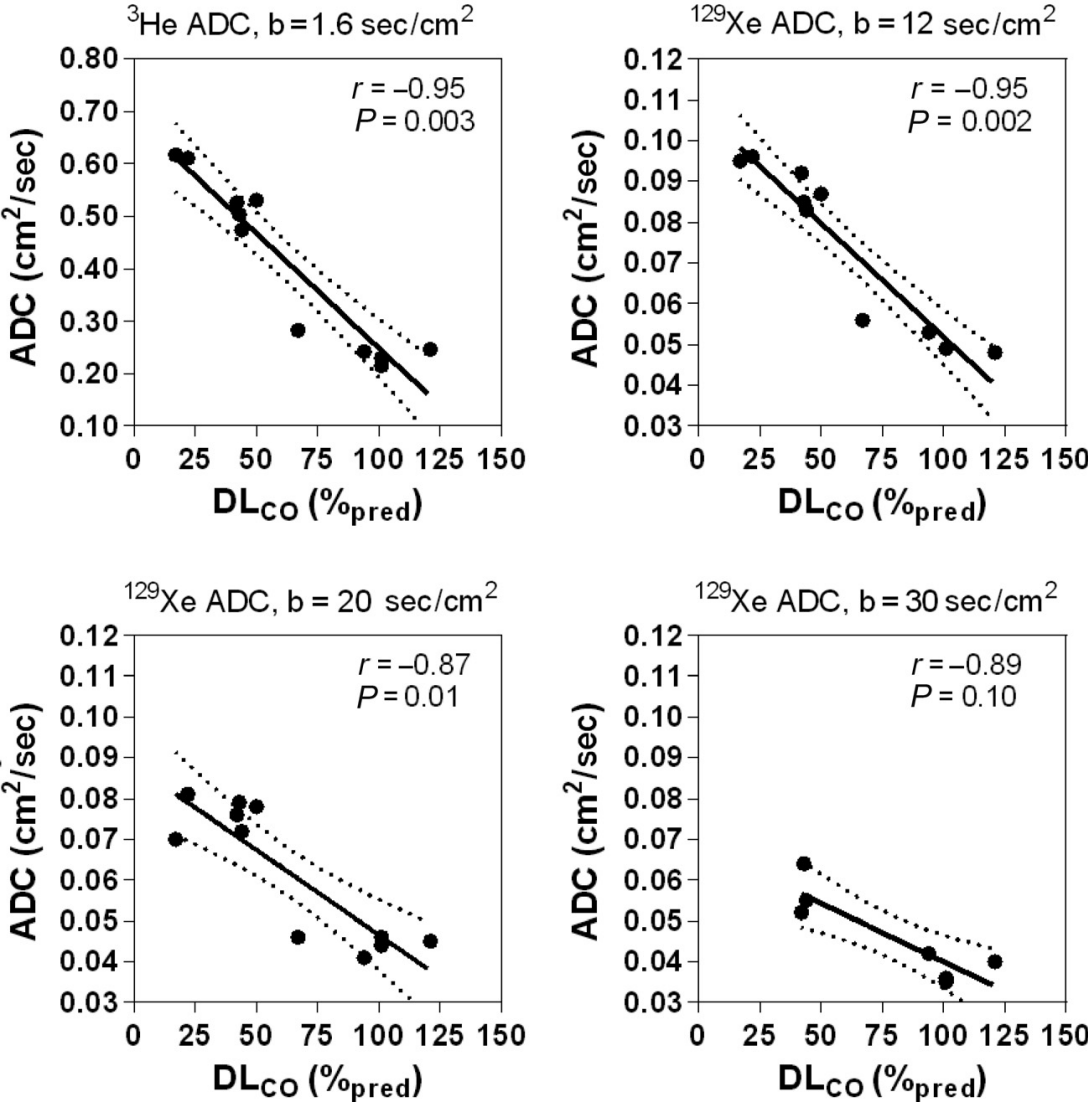
Correlations for ^3^He and ^129^Xe apparent diffusion coefficient (ADC) with DL_CO_ in all subjects. D_LCO_ was significantly correlated with ^3^He ADC (*r* = −0.95, *r*^2^ = 0.90, *P* < 0.0001, Holm–Bonferroni corrected *P* = 0.003, *y* = −0.0044*x *+ 0.69), ^129^Xe ADC_*b* = 12_ (*r* = −0.95, *r*^2^ = 0.90, *P* < 0.0001, Holm–Bonferroni corrected *P* = 0.002, *y* = −0.00056*x* + 0.11), ^129^Xe ADC_*b* = 20_ (*r* = −0.87, *r*^2^ = 0.76, *P* = 0.0005, Holm–Bonferroni corrected *P* = 0.01, *y* = −0.00042*x* + 0.088), and ^129^Xe ADC_*b* = 30_ (*r* = −0.89, *r*^2^ = 0.78, *P* = 0.008, Holm–Bonferroni corrected *P* = 0.10, *y* = −0.00029*x *+ 0.069). Dotted lines indicate the 95% limits of agreement.

Figure 5 shows Pearson correlations for DL_CO_ with ^3^He ADC, ^129^Xe ADC_b12_, ^129^Xe ADC_b20_, and ^129^Xe ADC_b30_ for all subjects. There were significant correlations for D_LCO_ with ^3^He ADC (*r* = −0.95, *P* = 0.003), ^129^Xe ADC_b12_ (*r* = −0.95, *P* = 0.002), and ^129^Xe ADC_b20_ (*r* = −0.87, *P* = 0.01), however, the correlation for DL_CO_ with ^129^Xe ADC_b30_ (*r* = −0.89, *P* = 0.008 uncorrected, *P* = 0.10 corrected) was no longer significant following Holm–Bonferroni correction.

The Pearson correlation coefficients between CT lung density thresholds (RA_950_, RA_910_, RA_856_, HU_15%_) and visual CT emphysema scores are shown in Table 3. Following Holm–Bonferroni correction, ^3^He ADC was significantly correlated with RA_950_ (*r* = 0.90, *P* = 0.008) and HU_15%_ (*r* = −0.92, *P* = 0.004); ^129^Xe ADC_b12_ was significantly correlated with RA_950_ (*r* = 0.85, *P* = 0.03) and HU_15%_ (*r* = −0.90, *P* = 0.008); ^129^Xe ADC_b20_ was significantly correlated with HU_15%_ (*r* = −0.86, *P* = 0.02); ^129^Xe ADC_b30_ was not significantly correlated with any CT emphysema measurements.

**Table 3.** Correlations for ^3^He and ^129^Xe apparent diffusion coefficient (ADC) with computed tomography emphysema measurements for chronic obstructive pulmonary disease ex‐smokers

	Pearson correlation coefficients *r* (p/p^1^)			
	^3^He ADC (cm^2^/sec)	^129^Xe ADC_b12_ (cm^2^/sec)	^129^Xe ADC_b20_ (cm^2^/sec)	^129^Xe ADC_b30_ (cm^2^/sec)
RA_950_ (HU)	0.90 (0.0004/0.008)	0.85 (0.002/0.03)	0.79 (0.006/0.09)	−0.49 (0.68/1.00)
RA_910_ (HU)	0.82 (0.004/0.06)	0.77 (0.009/0.11)	0.78 (0.008/0.11)	−0.06 (0.96/0.96)
RA_856_ (HU)	0.67 (0.04/0.32)	0.63 (0.05/0.35)	0.69 (0.03/0.27)	0.30 (0.81/1.00)
HU_15%_ (HU)	−0.92 (0.0002/0.004)	−0.90 (0.0004/0.008)	−0.86 (0.001/0.02)	0.44 (0.71/1.00)
Visual Score	0.79 (0.01/0.11)	0.76 (0.02/0.20)	0.66 (0.05/0.30)	−0.38 (0.75/1.00)

^1^ Holm–Bonferroni adjusted significance values.

Table 4 shows the results of multiple linear regression models with ^3^He ADC, ^129^Xe ADC_b12_, ^129^Xe ADC_b20_, and ^129^Xe ADC_b30_ as the predictor variables. In the multivariate regression model for DL_CO_ with ^3^He and all ^129^Xe ADC values included, ^129^Xe ADC_b12_ (*P* = 0.049) was the only significant predictor. In the multivariate regression model for RA_950_ with ^3^He and all ^129^Xe ADC values included, the only significant predictor was ^3^He ADC (*P* = 0.03). We also evaluated the multivariate regression model for HU_15%_, and although the overall model was statistically significant (*P* < 0.05), ^3^He ADC, ^129^Xe ADC_b12_, or ^129^Xe ADC_b20_ were not significant predictors.

**Table 4.** Multivariate linear regression model for DL_CO_ and computed tomography RA_950_ with ^3^He and ^129^Xe apparent diffusion coefficient (ADC)

Variable	Parameter estimate	Standard error	Fold change	*P*‐value
Model 1: DL_CO_				
^3^He ADC_b1.6_	663.85	218.63	3.04	0.09
^129^Xe ADC_b12_	−4974.20	1153.94	−4.31	**0.049**
^129^Xe ADC_b20_	−1165.39	795.22	−1.47	0.28
^129^Xe ADC_b30_	−935.27	539.89	−1.73	0.23
Model 2: RA_950_				
^3^He ADC_b1.6_	306.93	107.64	2.85	**0.03**
^129^Xe ADC_b12_	−1821.20	929.23	−1.96	0.10
^129^Xe ADC_b20_	292.72	370.07	0.79	0.46
^129^Xe ADC_b30_	–	–	–	–

Statistically significant *P*‐values (*P* < 0.05) are presented in bold.

## Discussion

The low and variable polarization levels and low diffusivity of ^129^Xe gas results in technical challenges for extracting physiologically meaningful pulmonary microstructural information based on ^129^Xe MRI. Moreover, although previous studies (Kaushik et al. 2011; Kirby et al. 2013, 2012d) reported ^129^Xe ADC based on a *b* value of 12 sec/cm^2^, it is still unclear how to provide the optimal balance between sensitivity to the lung microstructural abnormalities in emphysematous patients and feasibility with respect to image quality. Moreover, ^129^Xe MRI ADC generated using different *b* values may access different regions of the lung and likely probe different lung microstructure length scales thereby providing somewhat different spatial information relative to ^3^He ADC. For a better understanding of these measurements, we investigated the different ^129^Xe MRI ADC *b* values and their associations with anatomical and physiological measurements of COPD in a small but well‐characterized group of older never‐smokers and COPD ex‐smokers.

We observed (i) significantly greater ADC values for COPD ex‐smokers compared to older never‐smokers for ^3^He ADC, ^129^Xe ADC_b12_, and ADC_b20_, but not for ^129^Xe ADC_b30_, (ii) significantly greater anterior–posterior ADC differences in COPD ex‐smokers with emphysema compared to older never‐smokers for ^3^He ADC, ^129^Xe ADC_b12_, and ADC_b20_, but not for ^129^Xe ADC_b30_, (iii) significantly different mean ADC in the superior as compared to inferior lung regions between older never‐smokers and COPD ex‐smokers for ^3^He ADC, but not for ^129^Xe ADC, and finally, (iv) strong and significant correlations for ^3^He and ^129^Xe ADC with DL_CO_ and CT measurements of emphysema, but in a multivariate model, ^129^Xe ADC_b12_ was the only significant predictor of DL_CO_ and ^3^He ADC was the only significant predictor of CT measured RA_950_.

As expected, we observed significantly greater (worse) ADC for COPD ex‐smokers than for older never‐smokers for ^3^He, and as well for ^129^Xe ADC_b12_ and ADC_b20_, but not for ^129^Xe ADC_b30_. There was a large difference in mean ADC between subject subgroups for both ^3^He and ^129^Xe MRI, and this important finding suggested that both ^129^Xe MRI generated using *b* values of 12 and 20 sec/cm^2^ provide adequate sensitivity to lung microstructural abnormalities. These data are also in agreement with previous findings (Kaushik et al. 2011) that ^129^Xe ADC_b12_ can be used to distinguish between older never‐smokers and COPD subjects with emphysema. However, we must point out that ^129^Xe ADC_b30_ was not significantly different between subject subgroups and this may have been due to the low sample size as well as low SNR. For ADC_b30_, we demonstrated that the diffusion attenuation curves deviate from single exponential behavior after *b* = 20 sec/cm^2^, suggesting greater diffusion anisotropy (Ouriadov et al. 2013) compared to ADC_b12_ and ADC_b20_, and this greatly influences image quality, which was poor for ADC_b30_ images here. It is important to note that while SNR was not significantly different for ^129^Xe MRI acquired at all three *b* values, there was a significant correlation between image SNR and ADC for ^129^Xe ADC_b30_ only, suggesting that there may have been inadequate image quality to derive reliable ADC measurements. Clearly these are important considerations when optimizing and selecting pulse sequence parameters since image signal is expected to be reduced when the gradient amplitude is increased.

Second, there were statistically significantly greater AP_G_ for the COPD ex‐smokers with emphysema than the older never‐smokers for ^3^He ADC, ^129^Xe ADC_b12_, and ADC_b20_, but not for ^129^Xe ADC_b30_. DW MRI is sensitive to changes in the lung microstructure (Saam et al. 2000; Salerno et al. 2002) and is therefore an indirect measure of airspace size. Since the DW images were acquired in breath‐hold condition in the supine position, the measurement of the ADC on a slice‐by‐slice basis can be exploited to measure compression of the dependent lung due to gravity. Several studies have reported lower ADC values in the dependent (or posterior) lung slices relative to the nondependent anterior lung slices for ^3^He MRI (Fichele et al. 2004; Evans et al. 2007; Diaz et al. 2008) and ^129^Xe MRI with *b* = 12 sec/cm^2^ (Kaushik et al. 2011), which is expected due to compression of the lung parenchyma in healthy never‐smokers. However, in subjects with emphysema, the gradient in alveolar expansion in the gravity‐dependent direction is reduced, in part, because of gas trapping (Evans et al. 2007; Diaz et al. 2008). The finding that ^129^Xe ADC_b12_ anterior–posterior gradients were also reduced in the COPD ex‐smokers with emphysema in comparison to healthy never‐smokers is in agreement with these previous studies (Evans et al. 2007; Diaz et al. 2008; Kaushik et al. 2011). Furthermore, the finding that ^129^Xe ADC_b20_ anterior–posterior gradients were also reduced in the COPD ex‐smokers with emphysema in comparison to healthy never‐smokers suggests that ^129^Xe MRI with *b *=**20 sec/cm^2^ also provides a way to evaluate microstructural abnormalities.

Third, there was a significant difference in mean ADC in the superior as compared to inferior lung regions between the older never‐smokers and the COPD ex‐smokers with emphysema for ^3^He ADC, but not for any ^129^Xe ADC. It is important to note that in contrast with the results reported here, previous studies evaluating the ^129^Xe ADC SI gradients in older never‐smokers demonstrated elevated ADC values in the inferior lung regions (Sindile et al. 2007) or elevated ADC values the superior lung regions (Kaushik et al. 2011). It is clear that because of the lower diffusion coefficient of pure ^129^Xe gas, more heterogeneous gas mixing within the lung is likely to occur during image acquisition and this may, in part, explain the discrepancies in these previous reports. However, the ^129^Xe/^4^He gas mixture used in this study may have allowed more homogeneous mixing within the lung resulting in the lower SI gradient seen in the older never‐smokers, which is to be expected in healthy volunteers without emphysema. Although it is unclear why elevated ADC was demonstrated in the superior lung regions in comparison to the inferior lung regions for ^3^He MRI, but not for ^129^Xe MRI in the COPD ex‐smokers with emphysema, these differences may be explained by the different lung regions accessed by the gases, as shown in Figure 1, due to the different densities and viscosities of the gas mixtures. In this regard, we previously demonstrated significantly greater ventilation abnormalities for ^129^Xe MRI than ^3^He MRI in the same COPD ex‐smokers (Kirby et al. 2012d). In a regional evaluation of ^3^He and ^129^Xe ADC, we showed that mean ADC in lung regions accessed by ^3^He gas only were significantly greater than in the remaining lung (Kirby et al. 2013), suggesting that the more emphysematous lung regions filled more readily with ^3^He as compared to ^129^Xe gas. By extension therefore, if here the ^3^He gas more readily filled the centrilobular emphysematous regions within the upper lobe, this may explain the greater SI differences for ^3^He MRI. However, it is important to note that based on the estimated self‐diffusion coefficients for the ^3^He and ^129^Xe gas mixtures (0.211 and 0.826 cm^2^/sec for ^129^Xe/^4^He and ^3^He/N_2_, respectively; Kirby et al. 2012d), ^129^Xe gas is a better estimate of room air, which has an estimated self‐diffusion coefficient of 0.218 cm^2^/sec, as compared to the ^3^He gas mixture. Taken together this suggests that due to the different physical properties of ^129^Xe, the distribution of the ^129^Xe gas mixture that is imaged during a breath‐hold condition may more accurately reflect the regional distribution of oxygen gas mixed in room air as compared to ^3^He gas mixtures used during imaging.

Finally, we demonstrated strong and significant correlations for ^3^He ADC, ^129^Xe ADC_b12_, and ADC_b20_ with DL_CO_ and CT measurements of emphysema. DW gradients and diffusion time influence the measured ADC, and more importantly, they influence the spatial scale that the gas is probing (Woods et al. 2005). Therefore, ^3^He ADC and ^129^Xe ADC with different *b‐*values may provide different sensitivities to different lung microstructural environments and abnormalities. We investigated this by evaluating the relationship for DL_CO_ and CT lung density thresholds with ^3^He and ^129^Xe ADC. We observed strong correlations for ^3^He ADC, ^129^Xe ADC_b12_, and ADC_b20_, further suggesting that ^129^Xe DW imaging is sensitive to the same lung microstructural abnormalities as ^3^He ADC with *b* value 1.6 cm^2^/sec. However, we must also note that although strong and significant univariate correlations were observed for ^3^He and ^129^Xe ADC with DL_CO_, in the multivariate regression model ^129^Xe ADC_b12_ was the only significant predictor of DL_CO_. In contrast, ^3^He ADC was the only significant predictor of RA_950_. Although CT density‐threshold measurements correlate with pathology measurements of emphysema on resected lung specimens (Avni et al. 1996), it has been suggested that CT is not sensitive to subclinical or more mild emphysema (Muller et al. 1988; Miller et al. 1989). DL_CO_, on the other hand, despite low reproducibility, has been shown to be a very sensitive measure of early or mild subclinical emphysematous abnormalities. A recent study demonstrated elevated levels of endothelial microparticles in smokers with abnormally low DL_CO_ but normal spirometry (Gordon et al. 2011). Furthermore, in ex‐smokers without COPD, elevated ^3^He ADC, worse symptoms and exercise capacity were reported in subjects with abnormal as compared to normal DL_CO_ (Kirby et al. 2013). Since ^129^Xe gas has a lower diffusivity than ^3^He, we hypothesized that ^129^Xe MRI with greater diffusion weighting may be more sensitive to early or mild emphysema. The finding that ^129^Xe MRI was the only significant predictor of DL_CO_ lends support to this notion.

The diffusion time of 5 msec was selected for ^129^Xe MRI (Sukstanskii and Yablonskiy 2012; Boudreau et al. 2013) in order to probe a similar spatial length scale as ^3^He, and using this diffusion time, the characteristic diffusion lengths were approximately 0.49 and 0.46 mm for ^3^He and ^129^Xe, respectively. For these diffusion lengths, it is likely that both ^3^He and ^129^Xe gas atoms diffused beyond a single alveolus (diameter ~0.20 mm; Ochs et al. 2004) to a distal respiratory bronchiole (diameter ~0.40 mm; Horsfield and Cumming 1968). Although the majority of the COPD subjects evaluated in this study had moderate‐to‐severe emphysema, future studies, perhaps with improved polarization levels, should investigate the sensitivity of different ^129^Xe MRI imaging parameters, including different diffusion weighting and diffusion times, for evaluating emphysematous changes in subjects with more mild disease. Importantly, in our previous investigations we have demonstrated ^129^Xe MRI gas distribution measurements were more sensitive to airway wall abnormalities in asthmatics (Svenningsen et al. 2013) and to emphysematous bullae in COPD (Kirby et al. 2013). These findings suggest that due to the different physical properties of ^129^Xe gas compared to ^3^He gas, ^129^Xe may be more sensitive for the detection of airway obstruction and may therefore also be more sensitive for the detection of airway obstruction in patients with early or subclinical disease. However, this will need to be confirmed in future ^129^Xe MRI investigations.

We also believe that ^129^Xe MRI DW imaging may provide increased sensitivity for evaluating emphysematous changes in subjects with more mild disease. In this regard, we must acknowledge that ^129^Xe ADC_b30_ may not provide an appropriate estimate of emphysema in subjects with moderate‐to‐severe disease. In regions of the lung with significant emphysema, the ^129^Xe gas diffusion coefficient is likely strong enough to destroy the MRI signal in that region due to the greater ^129^Xe diffusion weighting, and the only signal remaining in the lung will be from the nonemphysematous lung microstructure. Therefore, the measured ADC values will reflect the healthier lung parenchyma, and not the emphysematous regions. However, in very early or mild subclinical disease, this may in fact be advantageous, as any small increase in airspace size will result in a reduction in signal intensity in the DW images. ^129^Xe ADC_b30_, due to the greater diffusion weighting may therefore provide greater sensitivity to mild microstructural changes than ^3^He ADC or ^129^Xe ADC_b12_, although this cannot be ascertained by the current study.

We recognize that this work was limited by several factors. First, the small number of subjects evaluated and in particular the small number of subjects in the ^129^Xe ADC_b30_ group, certainly limits the generalizability of these results. Another important limitation for both ^3^He and ^129^Xe ADC measurements is that these cannot be reported for the regions of the lung that are poorly or not ventilated. Such unventilated or poorly ventilated lung regions may be due to small airway occlusion, mucous plugs, airway wall thickening and inflammation, severe emphysema, or bullous disease. Previous studies (Kaushik et al. 2011; Kirby et al. 2012a) comparing ^3^He and ^129^Xe in COPD suggest that ^129^Xe gas distribution is sensitive to emphysematous bullae, and this is another consideration when performing ^129^Xe DW imaging in COPD subjects.

In summary, we evaluated ^129^Xe ADC *b* values of 12, 20, and 30 sec/cm^2^ in the same older never‐smokers and COPD ex‐smokers and compared these with ^3^He ADC, DL_CO_, and CT measurements of emphysema. We showed that ^3^He ADC and ^129^Xe ADC_b12_ or ADC_b20_ distinguished subjects with and without COPD. Although strong correlations for both ^3^He and ^129^Xe ADC were observed with DL_CO_ and CT measurements of emphysema, in a multivariate analysis ^129^Xe ADC_b12_ was the only significant predictor of DL_CO_. However, ^129^Xe ADC_b30_ may be appropriate for evaluating subclinical or mild emphysema, in this small group of older never‐smokers and ex‐smokers with moderate‐to‐severe emphysema, ^129^Xe ADC_b12_ provided a physiologically appropriate estimate of gas exchange abnormalities and alveolar microstructure, providing good evidence to support future ^129^Xe MRI studies of emphysema.

## Acknowledgments

We thank S. McKay and S. Blamires for subject recruitment, clinical coordination, and clinical database management; A. Wheatley for production and dispensing of ^3^He gas; and T. Szekeres for MRI of research volunteers.
